# Analysis of Metabolomics Datasets with High-Performance Computing and Metabolite Atlases

**DOI:** 10.3390/metabo5030431

**Published:** 2015-07-20

**Authors:** Yushu Yao, Terence Sun, Tony Wang, Oliver Ruebel, Trent Northen, Benjamin P. Bowen

**Affiliations:** 1 National Energy Research Scientific Computing Center (NERSC) and Computational Research Division, Lawrence Berkeley National Lab, Berkeley, CA 94720, USA; E-Mails: yyao@lbl.gov (Y.Y.); tsun1215@gmail.com (T.S.); tony.wang.95@gmail.com (T.W.); oruebel@lbl.gov (O.R.); 2 Life Sciences Division, Lawrence Berkeley National Lab, Berkeley, CA 94720, USA; E-Mail: trnorthen@lbl.gov

**Keywords:** SciDB, metabolite atlas, metabolomics, data analysis, IPython, Python, LC/MS, MS/MS, biology

## Abstract

Even with the widespread use of liquid chromatography mass spectrometry (LC/MS) based metabolomics, there are still a number of challenges facing this promising technique. Many, diverse experimental workflows exist; yet there is a lack of infrastructure and systems for tracking and sharing of information. Here, we describe the Metabolite Atlas framework and interface that provides highly-efficient, web-based access to raw mass spectrometry data in concert with assertions about chemicals detected to help address some of these challenges. This integration, by design, enables experimentalists to explore their raw data, specify and refine features annotations such that they can be leveraged for future experiments. Fast queries of the data through the web using SciDB, a parallelized database for high performance computing, make this process operate quickly. By using scripting containers, such as IPython or Jupyter, to analyze the data, scientists can utilize a wide variety of freely available graphing, statistics, and information management resources. In addition, the interfaces facilitate integration with systems biology tools to ultimately link metabolomics data with biological models.

## 1. Introduction

Data analysis is one of the grand challenges facing metabolomics research. There are many reasons for this, but most stem from the diverse physicochemical properties common to metabolites including solubility, ionization potential, and isomers. In comparison, detection of proteins, DNA, and RNA is much more straightforward. That is, where the latter are all biopolymers and can be directly identified based on well-defined fragmentation rules, each metabolite is largely its own puzzle [1]. Liquid chromatography coupled to electrospray ionization mass spectrometry (LC/MS) has become the most widely used metabolomics workflow as a result of its ability resolve complex mixtures of biomolecules [2].

The analysis of the LC/MS metabolomics often begins with defining metabolite features which are the combination of accurate mass and retention time. Unfortunately, both of these depend on many parameters that get defined as part of sample preparation and data acquisition. This dependence is because the chromatographic separation and elution is due to the choice of solvent and the biological matrix; in addition, desorption and ionization processes often generate artifacts and alter the types of ions detected. For example, a sugar that is detected as an ammonium ion adduct in one sample may be largely found as a protonated species in another sample. Similarly, the retention time for a given compound depends on the chromatography conditions. This high degree of dependence on the exact experimental conditions and samples is a major confounding factor in large-scale metabolomic experiments. Not only does this dependence on the parameters cause inconsistencies across experiments, it also must be communicated with the dataset it produced.

The tight coupling between metabolomics observations, sample preparation, and experimental parameters has long been recognized as a critical challenge facing the metabolomics community, most notably in a series of publications on the metabolomics standards initiative [3,4,5]. While there is broad consensus regarding the need for standardization of workflows and data analysis, this need remains unmet. This is especially problematic for *untargeted* metabolomics experiment, the unbiased analysis of the data to identify changes in features prior to metabolite identification. For advancement of our understanding of biochemical networks, unbiased analysis is very appealing because it does not presuppose an understanding of the metabolism *a priori*. However, despite major efforts, unbiased profiling is much less popular than targeted workflows. We can speculate that the difficulty in data analysis is one of the reasons. While matrix effects can still bias these, the data analysis is more straightforward. Specifically targeted workflows use authentic standards to optimize specific extraction, chromatographic, and mass spectrometry methods to measure metabolites of interest. In comparison, identifying whether an observation in an untargeted workflow is real or an artifact due to ion-suppression, salt effects, or other confounding aspects is very difficult, often making what should be simple tasks into significant bottlenecks. There are several recently described workflow-tools and data processing tools that aim to achieve the primary analysis of untargeted metabolomics data [6,7,8,9].

Several years ago we proposed the Metabolite Atlas concept as a mechanism for dealing with the unknown complexity associated with untargeted metabolomics experiments [10]. Recognizing that while a wide range of powerful algorithms exist for comparing and annotating features in mass spectrometry data [11,12]. This information isn’t often effectively leveraged for future experiments. By tracking feature annotations in method and sample specific Metabolite Atlases this information reused it for experiments; similar to the SetupX and BinBase system which is widely used in gas-chromatography/mass spectrometry (GC/MS) based metabolomics [13]. This concept is very simple and essentially makes untargeted experiments behave more similarly to targeted experiments by leveraging the characteristics that describe compounds detected under the exact same experimental conditions that have been applied to similar samples. By being “method and sample-specific” Atlases developed based on observations for a specific sample and LC/MS method would be used primarily for new data acquired for a very similar sample and LC/MS method. However this requires the development computational infrastructure for accessing vast amounts of raw mass spectrometry data, tracking metadata about experimental descriptions, and specifications of metabolite feature annotations [14].

Here we present the computational infrastructure for Metabolite Atlases. This is based on the use of IPython and Jupyter notebooks as an interface for data analysis and construction of method and sample specific metabolite atlases using the online metabolite atlas database [15]. Similar to targeted analyses an Atlas is used to extract the metabolite features from experimental data files using specified constraints based on chromatographic and mass spectrometric parameters. Users are able to visualize metabolite features within the retention time windows defined in the Atlas and can adjust retention times as needed. Since all of the analysis is performed within the IPython and Jupyter notebooks, the extensive Python libraries available for scientific computing can be used to perform advanced analysis on the resulting data tables.

## 2. Methods

Metabolite Atlases are specific for a sample-type and a chromatography method and define the m/z and retention time bounds for specific compounds [10]. Once these are defined, the raw data can be processed using this information to extract peak areas for compounds of interest in each file within an experiment. This is a contrast to conventional untargeted metabolomics workflows that start with feature extraction and comparison and identification is only performed on a small, select set of features.

**Metabolite Atlas SciDB data layout**. Due to the scale, complexity, and multidimensional nature of LC/MS data, high performance computing is necessary to quickly perform data access. High resolution mass spectrometers have full profile spectra that must be digitized into hundreds of millions of mass bins to properly preserve all the necessary data. However, vendors of these instruments are performing a two-step data reduction to compensate for the data size escalation. First, m/z values with low signal intensity are removed; and second, identified peaks are stored only as their m/z centroid and the intensity at that m/z. This is leading to a trend where file sizes are actually on a downward trend due to these advances in compression. The SciDB database service hosted at NERSC has been described previously and is used by Metabolite Atlas for raw data storage and access [16].

Multiple steps are required to load data into SciDB/Metabolite Atlas. First, it is necessary to convert raw LC/MS data from proprietary vendor supplied formats into the mzML open source format with the msconvert application provided as part of the Proteowizard package [17,18]. When prompted by a user, the mzML files are parsed with pymzML and loaded into a SciDB array.

Operations are written in SciDB to select data points based on the following parameters: m/z, retention time, intensity, ms-level, polarity, precursor ion m/z, precursor ion intensity, collision energy and file id. For selecting spectra and chromatograms, two operations are required. First, raw data points are sliced from the 9-dimensional array based on user-supplied ranges. Second, the data points are aggregated and put into a histogram on either a m/z or time axis. As shown previously, these operations can be performed quickly using the SciDB application [16].

**Data Management.** In Metabolite Atlas we need to handle two types of data: the raw data in mzML format, and the metadata. Each run in Metabolite Atlas corresponds one mzML file. Each mzML file is loaded into SciDB as a slice of a multi-dimensional array. Once loaded, the mzML files are backed up to tape storage. Metadata includes experimental descriptions, sample descriptions and Metabolite Atlases. Due to the unstructured nature of this data, MongoDB is used.

**Integrated web services.** Via the web, simple requests enable the querying of LC/MS data, sample metadata, experiment metadata, and descriptions of the compounds observed in an experiment. The Django Python package makes building web requests that integrated diverse data stores straightforward [19] The API defined in Django allows access to both of these resources.

**Web based application programming interface (API) description.** The commands and their required parameters, shown below in Table 1, are used to get chromatograms, bounds for metabolite features, and update them. As is described above, in SciDB, collision energy is stored for ions of ms-level greater than one. For those ions, the precursor m/z, collision energy, and precursor ion intensity are stored. In the future, queries will be developed that utilize these parameters.

**Table 1:** Integrated metabolite atlas API for simultaneously querying raw data along with compound specifications.

Method	URL	Options	Description
GET	/run/	{“L”:<level>, “P”:polarity, “arrayname”:<myArray>, “fileidlist”:<myList>, “max_mz”:<mzMax>, “min_mz”:<mzMin>, “min_rt”:<rtMin>, “max_rt”:<rtMax>, “nsteps”:<2000>, “queryType”:”XICofFile_mf”} JSON	Gets chromatograms for a given mz and rt specification for one or more files.
GET	/api/dict/<dict_id>/		Gets details about a specified compound dictionary
PUT	/api/dict/<dict_id>/	{“<field_name>”: <field_val> ...} JSON	Completely replaces the compound dictionary fields with the JSON object
GET	/api/compound/<compound_id>/		Gets details about a specified compound
PATCH	/api/compound/<compound_id>/	{“<field_name>”: <field_val> ..., “removed_fields”: [...list of removed field names...]} JSON	Updates the compound fields with the specified values
PUT	/api/compound/<compound_id>/	{“<field_name>”: <field_val>...} JSON	Completely replaces the compound fields with the JSON object

## 3. Results and Discussion

LC/MS based metabolomics is a rapidly growing field that is being applied to an ever-increasing diversity of samples using an ever-increasing diversity of experimental workflows. Thus, interpretation of metabolomics data must be a multidisciplinary effort. As a result of this diversity there are many experimental workflows for acquiring information about the metabolome and there is a need for computational systems for sharing and tracking this information. This need was described long ago where the need to track and share metadata about a metabolomics experiment was recognized.

We have developed a framework and interface for storing raw LC/MS and MS/MS data. This framework allows queries to operate on the raw data and return selections based on m/z and retention time for files of interest. In addition, this framework makes it transparent and straightforward to implement algorithms that operate on these selections of data. Most importantly, this framework provides unification of the data exploration and analysis with the chemical identifications. Often, it is a time consuming challenging process to identify significant number of metabolites in an untargeted metabolomics workflow.

**Figure 1:**
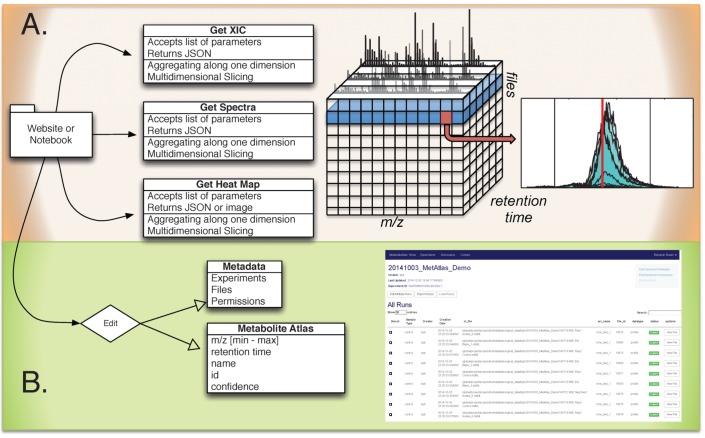
Overview of Metabolite Atlas implementation scheme. (**A**) raw data extraction of chromatograms and spectra from a large number of LC/MS runs is facilitated by high-performance computing applications such as SciDB; (**B**) the specification, update, and management of metadata about experiments, samples, and of compounds in a Metabolite Atlas is facilitated by standard database applications such as MongoDB. The integrated analysis of these components via web-based interfaces makes the analysis and sharing of experimental observations in the context of raw data possible.

Shown in Figure 1 is the overall breakdown of components in the Metabolite Atlas framework and its associated interfaces. Raw mass spectrometry data is captured in a SciDB database running on several nodes of a cluster at NERSC. From a web-client or programmatic API, queries to this database make it possible to get spectra, chromatograms, or other subsections of the raw data that have been aggregated along a specific dimension. These selection operations are the most commonly used operations for exploring and analyzing mass spectrometry data. As described recently, by using a high-performance computing application like SciDB, these operations can be made in a timely manner [16]. Due to NERSC security policies public access for [20] is not available at the time of publication. However, potential users can request access to the system by obtaining a NERSC account. Users with activated accounts will be able to use the Metabolite Atlas framework including IPython notebooks, file conversion, file transfer, and analysis creating and sharing. Most users take a short online course from Code Academy or Coursera to learn the basics of Python programming to ease their transition into Metabolite Atlas. 

The speed of the operations makes it possible to make many considerations about the data in a short amount of time. At each moment, the experimental scientist can capture their annotations as structured “Metabolite Atlas” metadata. At the simplest implementation, the Atlas captures the assertion that “Adenine” is observed at “9.4” min with an “m/z” of 1234 in a particular file. The specification of this compound identification in a structured way facilitates propagation and sharing in ways that were difficult and not reproducible before.

**Figure 2:**
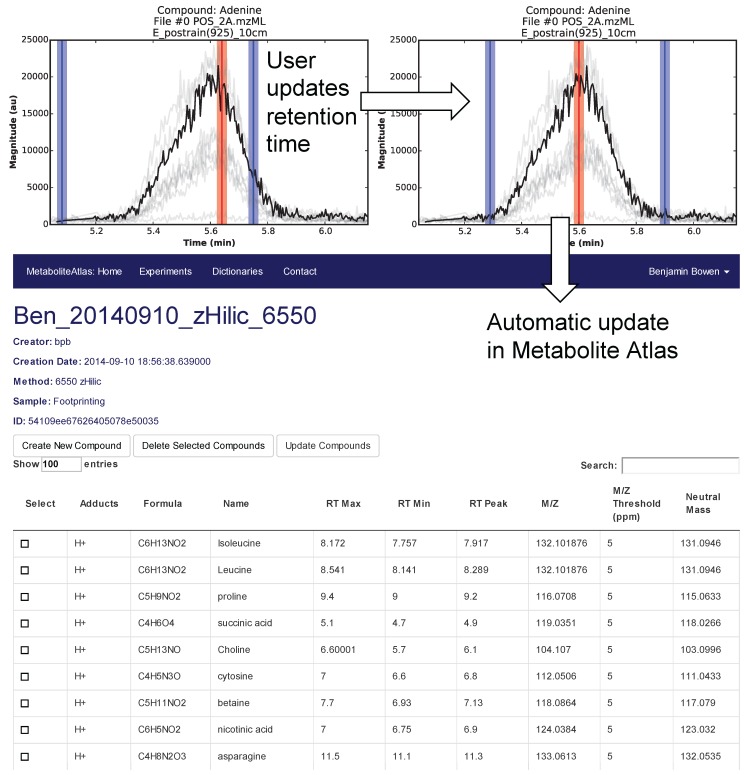
User interface for adjusting the retention time bounds. Integrated access to raw LC/MS data and a Metabolite Atlas is used to adjust retention time bounds. As improved retention and m/z bounds are specified the parameters for each compound are automatically updated in a Metabolite Atlas.

Selection of appropriate retention times is a critical and often time-consuming process. To facilitate this process Metabolite Atlas has a user interface enabling direct adjustment to retention time bounds as shown in Figure 2, for the example of nicotinamide. In this case, the retention time bounds for nicotinamide are observed to not precisely conform to the actual measured retention time characteristics of the measured chromatogram for nicotinamide. Based on this observation, the user updates their Atlas for nicotinamide and the results are automatically updated in the Metabolite Atlas.

The framework also facilitates storing and sharing descriptions about samples and experiments in a structured way. This is essential for propagation of identifications in an LCMS experiment. A peak identified as “*Compound X*” in one sample prepared with a specific extraction, reconstitution, and chromatography method might appear identical to another compound from a different sample or prepared using a different method. Thus, sample and method specific constraints on Metabolite Atlases make sharing of compound IDs have a rational. Once the form of the molecular ion is identified, *in silico* identification strategies including MIDAS and MetFrag provide effective strategies for compound identification [21,22].

This is important because many people with the same goals can’t leverage the work of the community. The majority of effort comprehensively understanding metabolomics data is associated with putative identifications of unknowns. The degeneracy associated with adducts, ionization, isotopes, in source degradation, ion suppression, saturation of detectors, chromatographic artifacts, unknown stereo-isomers and structural isomers has led to an explosion of challenges for reliable interpretation of metabolomics. This has led to a lack of clear expected deliverables from metabolomics workflow.

Many other research teams have described the need for or are currently working on allied topics to our Metabolite Atlas framework. Early work focused on capturing in a structured vocabulary the description of experimental parameters and concisely represent the results [23]. Several data warehousing solutions are available for mass spectrometry where raw data files along with descriptions of the sample preparation and acquisition are publicly available. The most recent of these, *Metabolights*, is providing high quality descriptions of the parameters describing the acquisition [24]. At this time, more than 100 experiments have been contributed to *Metabolights*; MassBank provides a portal for user viewing and submission of spectra for pure compounds [25]; and workflow tools allow users to contribute code that performs atomic-operations and chain them together [6,26]. In addition to schemas, data-warehousing and workflows, important algorithms are having a big impact overcoming the naïve oversimplification of spectral similarity algorithms and that of molecular weight assignment given a complex spectrum of adducts [27,28]. In comparison to all the above strategies, the Metabolite Atlas framework enables remote access to high-performance computing resources using a user friendly narrative-notebook interface for extremely high-speed queries to raw data that enable user-designed workflows while simultaneously tracking the metadata about experimental parameters and compounds detected [29].

**Figure 3:**
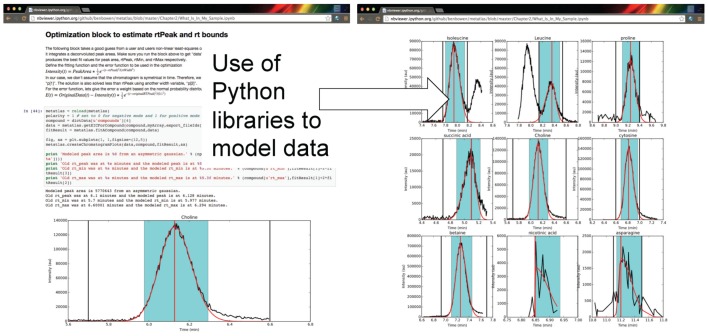
Authenticated users can acquire data from Metabolite Atlas using IPython and Jupyter notebooks. These notebooks provide a user friendly interface to the Python programming language which contains extensive libraries for data processing including peak fitting as shown here. These notebooks can be easily shared via the nbviewer service [32]. Typical notebooks contain code for analysis, results, and text explaining the purpose of the code.

The framework for computing and analysis is made accessible via a user interface which can capture the steps of an analysis from raw data, statistical analysis, and visualization in transparent and shareable format. Shown in Figure 3 are examples of the Metabolite Atlas accessed through an IPython and Jupyter notebook web interface. The use of these narrative notebooks allows users to share findings and methods through public repositories such as github [30]. As has been shown numerous times, popular methods in social networks will become widespread [31]. These methods will likely reduce the burden on analysis for the degenerate features detected in LC/MS experiments.

**Figure 4:**
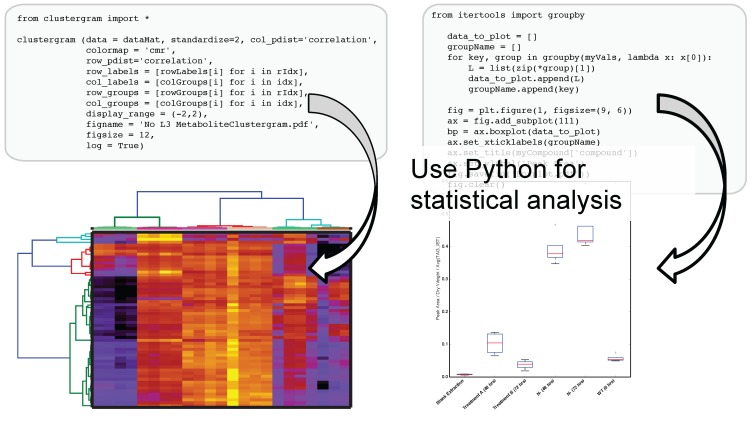
After optimizing the bounds for an Atlas, a user can acquire peak areas from Metabolite Atlas and perform statistical analysis for the compounds detected in their experiment. Python’s scientific libraries for statistical analysis can easily be implemented to perform common analysis such as hierarchical clustering and statistical confidence testing. Development of peak-shape modeling tools will be an important next step to deal with low-intensity peaks and missing values.

The use of IPython and Jupyter notebooks is not unique to Metabolite Atlas. They are the fastest growing application of any programmatic interface today. This gives users of Metabolite Atlas access to algorithms for clustering through the SciPy and Scikit stats models. Factorization of data into component parts through NumPy and SciPy. As can be seen in Figure 4, this integration with these powerful toolkits enables the user to make graphical outputs using Matplotlib and other visualization packages as well as perform routine statistical tests. Through the Python programming language and the linkages to bind the R programming language through the IPython interface users can create custom analysis. Although plotting, factorization, and clustering are specifically called out above, analysis ranging from compound-substructure searching, N-degrees of freedom statistical testing, multiparameter optimization are all at hand, and given the low-barrier to entry of the IPython notebook interface to the novice programmer, user-defined analysis are easily built to suite the needs of each experiment.

## 4. Conclusion

In conclusion we have described a computation framework for creating, sharing and updating Metabolite Atlases and interfacing them with IPython and Jupyter notebooks for data analysis. Adoption of this framework can provide transparency to data management and simplify workflows. Particularly exciting is the integration of metabolomics data with other systems biology data. We anticipate that Metabolite Atlases can be compared alongside genomes for gene annotations. Measured metabolites can be compared to those predicted in a COMPOUNDS.DAT file from a Pathway/Genome Database (PDGB) determined for a genome using Pathway Tools [1,33]. Using the KBase Model Building tools, measured metabolites can be compared to predictions of genome function and flux balance models [34].
